# The Institutional Worker

**Published:** 1917-03-24

**Authors:** 


### INSTITUTIONAL WORKER

**Being a Special Supplement to "The Hospital"**

### OUR BUREAU OF INFORMATION.

### Rules for Correspondents.

1. Every letter must be accompanied by the coupon to be cut from the back cover (inside page) of The Hospital, current issue, and must contain the name and address of the correspondent with pseudonym for publication if desired. Replies by post cannot be given save under exceptional circumstances at the Editor's discretion.

2. Letters from Approved Homes in reply to special needs published in the Bureau should state terms and full particulars, and be sent prepaid under cover to the Editor of the Bureau with name written across coupon for identification.

3. Proprietors of Homes which have not yet been entered on the List of Approved Homes, but have spare accommodation likely to suit special needs, are invited to write for an application form for registration. The fee for registration, which includes two announcements of the Home in the Bureau and other privileges, is 10s.

4. All communications to be addressed to the Editor of The Hospital, 28 Southampton Street, Strand, London, W.C.2, and marked "Bureau of Information."

### INSTITUTION FACTS AND FIGURES.

### The Service of Patients' Dinners.

### Answers to February Question.

The question was:

**What is the best method of serving patients' dinners so as to ensure them reaching the patient perfectly hot and appetising?**

### By "Pen."

Anyone who has adopted the profession of sick nursing realises that one of the most important factors in the progress towards recovery is that of appetisingly served food.

Supposing there are several wards to be supplied, each containing between twenty and thirty beds, it is obvious that as little time as possible must elapse between dishing-up and serving. The dinners are generally cooked in one large kitchen, which should communicate with the various floors by means of a lift. Large metal boxes, each containing a set of boxes labelled "Meat," "Vegetables," etc., should be provided. The large boxes should have an inside lining, and between the two walls should be a cavity which can be filled with boiling water just before the dishing-up process begins. The small boxes should be warmed and filled and covered up immediately with their well-fitting lids. As soon as several sets of tins have been filled they should be packed inside the large tins, and dispatched on a trolley to their respective wards. The lift used for this purpose should not be used for anything else during the dinner hour. The cook, in the meantime, proceeds to fill the remainder of the boxes, ready for dispatch when the trolley returns.

In the wards certain arrangements should be made to facilitate the speedy distribution of the meal. The plates should be well warmed in the oven of the ward kitchen, trays and tables should be laid in readiness, bread and drink handed round, and the patients comfortably propped up ready to start their meal as soon as they receive it. Finally, all the nurses and wardmaids should be ready to give round the filled plates as soon as the sister has served the food from the hot tins. The condiments are generally put on the plates, in a large ward, to save the time which would be taken up in running to each bed with a cruet. The individual tastes of the patients must be considered when serving, as it is no use sending an enormous plateful of food to a patient whose appetite requires a little tempting, and the most scrupulous attention must be paid to the crockery and glass.

In a small hospital, where the dinners are served from the kitchen where they have been cooked, there should be no more difficulty experienced in serving hot meals than in a private house; but if expense is not the chief object, it is a great boon to have hot-water plates with covers, on which the food can be kept hot for a considerable time.

### By "Housekeeper."

In the majority of hospitals the serving of patients' meals seems to be a difficult problem, and it is surprising how many complaints arise from this source, and often they are justified.

I have seen various methods for serving out meals, but the one I consider best is in practice at an up-to-date, well-organised hospital of six hundred beds, that has over three hundred military patients.

Special dinner tins are used for carrying the dinners. The bottom part is constructed to be filled with boiling water for heating the tin, and the upper part is divided into three portions, one for meat, the other two for vegetables; a tight-fitting lid completes the tin. For special diets similar tins are used, with smaller partitions.

The meat, instead of being sent uncarved to the ward, is cut up on the steam heater in the general kitchen; this has the advantage of keeping the meat hot, which is impossible when carved on an ordinary dish. While the meat is being carved an assistant puts in the vegetables; the tin is put on the trolley, and taken to the ward. Twenty minutes is the time allowed to serve out the dinners for nearly six hundred patients. If the joint is sent to the ward for carving, a metal meat-dish, made to be heated with hot water, is useful to keep the meat hot while carving.

At this hospital it has been proved to be more economical to carve in the general kitchen; the bones are kept for stock, and there is less waste. Nurses, as a rule, are not good carvers.

The ward staff must co-operate with the kitchen staff if the arrangements are to be carried out to perfection. Punctuality in both departments must be the rule; the ward staff should be ready to attend to the dinners the moment they arrive, and the sister in charge of the ward should see:

1. That the nurses do not begin dressings, etc., that they cannot finish before meal-times.
2. Knives, forks, and spoons should be ready beforehand.
3. Food should not be served by the open window.
4. Plates should be well heated.
5. Trays for bed patients should be set beforehand.
6. Cold food should be put on the trays before the hot.
7. Trays should be carried to the patients as soon as ready, and not allowed to wait until all have been prepared.
8. If the pudding is a hot one, it should not be served at the same time as the meat.
9. Dinners intended for helpless patients that the nurse has to feed should be prepared last.

Special diets are usually sent out of the kitchen first, and should be served at once, so that no mistakes can be made, also to give time to attend to the ordinary meals.

If possible, the head nurse should supervise the giving out of meals, and see that all her available staff are at hand to assist.

### QUESTIONS FOR MARCH.

(1) PREVENTION OF WASTE IN FOODSTUFFS.

In view of the necessity for economising food supplies, give comprehensive suggestions for enabling the housekeeper to prevent the waste of foodstuffs in her institution.

(2) OUTFIT FOR FACTORY MEDICINE CUPBOARD.

Name the items for a complete outfit for a factory medicine cupboard for the use of the Welfare supervisor. Besides every kind of accident, a large number of minor ailments are dealt with daily.

RULES.

The following rules must be observed :—

1. Contributions must be written on one side of the paper. Brevity and terseness are desirable features. The MSS. must bear the name and address of the sender and be accompanied by coupon to be cut from the back cover (inside page) of the current issue of The Hospital. A pseudonym must be chosen if the name is not to be published.

2. Contributions must be addressed to the Editor of The Hospital, Institutional Worker Supplement, 28 & 29 Southampton Street, Strand, London, W.C. 2, should reach him before the end of the current month, and be marked in the left-hand corner "Facts and Figures."

**A minimum payment of Half-a-Guinea will be made for each published answer.**

### QUESTIONS INVITED.

In connection with our Question Box, we offer every month five shillings each for the two best questions which are sent in for consideration. The questions may be on any institutional subject and concern any department, from the secretary's to the porter's, or the matron's to the domestic staff; the one essential is that they must be practical and deal with points that have a definite relation to hospital or institutional administration, upkeep, finance, or management. Points relating to artificers' work, housekeeping, the laundry, out-patients, and other practical matters will be welcome. It is hoped that thus, when difficult or doubtful points arise in the course of their work, institutional workers will be encouraged to put them in the form of a question and send them to the Editor, The Hospital Bureau of Information, 28 & 29 Southampton Street, Strand, W.C. 2, marked "Question Box."

The best questions will be published in due course and our readers will be given the opportunity of answering them. Thus all institutional workers may be able to co-operate with the view of helping the work and smoothing the difficulties of each other. In short, we want the Question Box of the *Institutional Worker* to be freely used by every worker habitually.

### ENQUIRIES AND ANSWERS.

### *THE SICK AND IN NEED.*

**Nursery Hospital for Child.**

You might possibly gain admission for your child to Miss Moyra Hill Trevor's Nursery Hospital, Brynkinalt Cottage, Chirk. Children under ten years of age are admitted free; between ten and fifteen years old a payment of 3s. a week is required (only surgical cases being taken at these ages). Write to the Matron for advice in the first instance.—*Mother.*

**Convalescent Home for Child of Two Years.**

Write to the Hon. Secretary, St. Luke's Home for Children, Woodley, Reading. Children of two years are admitted to this Home upon payment of 7s. 6d. per week. There is no limit to the duration of stay. London cases and open wounds are admitted. —G. L. D.

### *WAR MATTERS.*

**Sphagnum Moss for Surgical Dressings.**

You possibly mean the sphagnum moss which is so largely used now for surgical dressings. If you write to the Hon. Secretary of the Edinburgh War Dressings Supply Committee, 37 Palmerston Place, Edinburgh, you will, we feel sure, gain all the information you require. The moss, when gathered, has to be picked clean, free of sticks and leaves, prepared in various ways, and enclosed in muslin bags before being sent out. A note appeared upon this subject in these columns in the issue of December 30, 1916.—H. M. P.

### *THE HOUSEKEEPERS' DEPARTMENT.*

**Boiling Potatoes in their Skin.**

It is a good plan when cooking potatoes in their skin to boil them for five minutes (after scrubbing them thoroughly) and then pour away the water and fill up the saucepan with a second supply of water and salt. This helps to clean the skins more thoroughly.—Paddy.

**Supper or High-Tea Dish.**

The following makes a nice dish for supper or high-tea: Break an egg (as for poaching) into a small dish (shell-shaped or round are the nicest); add a little margarine and grated cheese and place in a medium oven. It should be served in the dish in which it is cooked.—M. A. C.

### *MISCELLANEOUS.*

**Pension Fund Policies and War Loan.**

You should make all inquiries regarding your policy to the Secretary, Royal National Pension Fund for Nurses, 15 Buckingham Street, Strand, W.C.—Member.

### *EMPLOYMENT AND TRAINING.*

**Institution for Private Nurses at Newcastle-on-Tyne.**

You might apply to the Lady Superintendent, Nurses' Home, 2 Granville Road, Newcastle-on-Tyne. Nurses holding a certificate of three years' training from a recognised training school are accepted, after a personal interview if possible, for three months' trial. Nurses receive four weeks' holiday in the year; a salary of £30 to £34 is given, and the profits of the institution are distributed among the nurses in the form of bonuses, and are apportioned according to the number of weeks a nurse has been at work in the year. Board, lodging, laundry (when not at cases), and in- and out-door uniforms are provided.—Beryl.

**Tuition in Natural Physical Methods.**

We have no doubt that you would find it possible to obtain instruction in the use of Zander machines and other physical apparatus in London. Some of the larger military hospitals are now well equipped with such apparatus. Possibly if you applied to Dr. Hamel, of 1 Stratford Place, London, W., who has a complete gymnasium equipped with many types of electro-medical and other physical apparatus, he would help you in the matter.—Nurse B.

**East London Nursing Society.**

The office address of the East London Nursing Society is Charterhouse, London, E.C. This Society was formed to nurse the sick poor of East London in their own homes, by means of trained nurses, and is affiliated to the Queen's Jubilee Institute. Nurses must be fully trained in all branches of nursing, and possess the certificate of a recognised training school. The salary is £52 rising to £56 per annum, with lodging and uniform and all necessary working appliances.—Thistle.

**Special Training during Waiting Period.**

You might usefully fill up your time whilst waiting to be admitted into a large nursing school by gaining some practical instruction in the nursing of sick children at the Invalid Children's Convalescent Nursing Home, Winifred House, Wray Crescent, Tollington Park, London, N. Applicants are received for one or two years' training; certificates are not granted, but testimonials of efficiency are given upon satisfactorily completing engagement. If considered suitable, the probationers are recommended to hospital authorities for training in the larger schools. —Anxious to Know.

